# Contribution of hypothalamic orexin (hypocretin) circuits to pathologies of motivation

**DOI:** 10.1111/bph.17325

**Published:** 2024-09-24

**Authors:** Aida Mohammadkhani, Caitlin Mitchell, Morgan H. James, Stephanie L. Borgland, Christopher V. Dayas

**Affiliations:** 1 Department of Physiology and Pharmacology, Hotchkiss Brain Institute, The University of Calgary, Calgary, Alberta, Canada; 2 School of Biomedical Sciences and Pharmacy, University of Newcastle, University Drive, Callaghan, New South Wales, Australia; 3 The Hunter Medical Research, New Lambton Heights, New South Wales, Australia; 4 Department of Psychiatry and Brain Health Institute, Robert Wood Johnson Medical School, Rutgers University, Piscataway, New Jersey, USA

**Keywords:** addiction, dopamine, eating, orexin hypocretin, orexin reserve, mesolimbic system, nucleus accumbens, paraventricular nucleus, ventral tegmental area

## Abstract

The orexin (also known as hypocretin) system, consisting of neuropeptides orexin-A and orexin-B, was discovered over 25 years ago and was immediately identified as a central regulator of sleep and wakefulness. These peptides interact with two G-protein coupled receptors, orexin 1 (OX_1_) and orexin 2 (OX_2_) receptors which are capable of coupling to all heterotrimeric G-protein subfamilies, but primarily transduce increases in calcium signalling. Orexin neurons are regulated by a variety of transmitter systems and environmental stimuli that signal reward availability, including food and drug related cues. Orexin neurons are also activated by anticipation, stress, cues predicting motivationally relevant information, including those predicting drugs of abuse, and engage neuromodulatory systems, including dopamine neurons of the ventral tegmental area (VTA) to respond to these signals. As such, orexin neurons have been characterized as motivational activators that coordinate a range of functions, including feeding and arousal, that allow the individual to respond to motivationally relevant information, critical for survival. This review focuses on the role of orexins in appetitive motivation and highlights a role for these neuropeptides in pathologies characterized by inappropriately high levels of motivated arousal (overeating, anxiety and substance use disorders) versus those in which motivation is impaired (depression).

## 1 | OREXIN NEUROPEPTIDES AS MOTIVATIONAL ACTIVATORS

Motivation is required for energizing adaptive behaviour that is critical to survival. Over the past two decades, the brain orexin-A and -B (also referred to as hypocretin-1 and −2) system has become a major focus of efforts to identify the neurobiological basis of motivation. Prepro-orexin mRNA is expressed exclusively by neurons in the lateral hypothalamic (LH) area, which includes the perifornical area (PFA), lateral (LH) and dorsomedial (DMH) nuclei of the hypothalamus (de Lecea et al., 1998; Sakurai et al., 1998). Prepro-orexin mRNA encodes a single polypeptide, prepro-orexin, that undergoes proteolytic cleavage to form orexin-A, a 33-amino acid residue peptide with two intramolecular disulfide bridges in its N-terminal domain, and orexin-B, a 28-amino acid residue linear peptide. Orexin-A and orexin-B share 46% sequence homology (Heifetz et al., 2013; Peyron et al., 1998; Sakurai et al., 1998). The orexin peptides act on two G-protein-coupled receptors (GPCRs), orexin 1 (OX_1_) and orexin 2 (OX_2_) receptors, that are widely distributed throughout the brain. Orexin-A has an equally high affinity to OX_1_ and OX_2_ receptors, whereas orexin-B displays ~10-fold selectivity for OX_2_ receptors (Nagahara et al., 2015; Sakurai et al., 1998). Orexin receptor activation stimulates intracellular calcium mobilization and other effector systems, followed by the modulation of various downstream-signalling pathways (reviewed in Kukkonen & Leonard, 2014) resulting in a neuroexcitatory activity.

**FIGURE 1:**
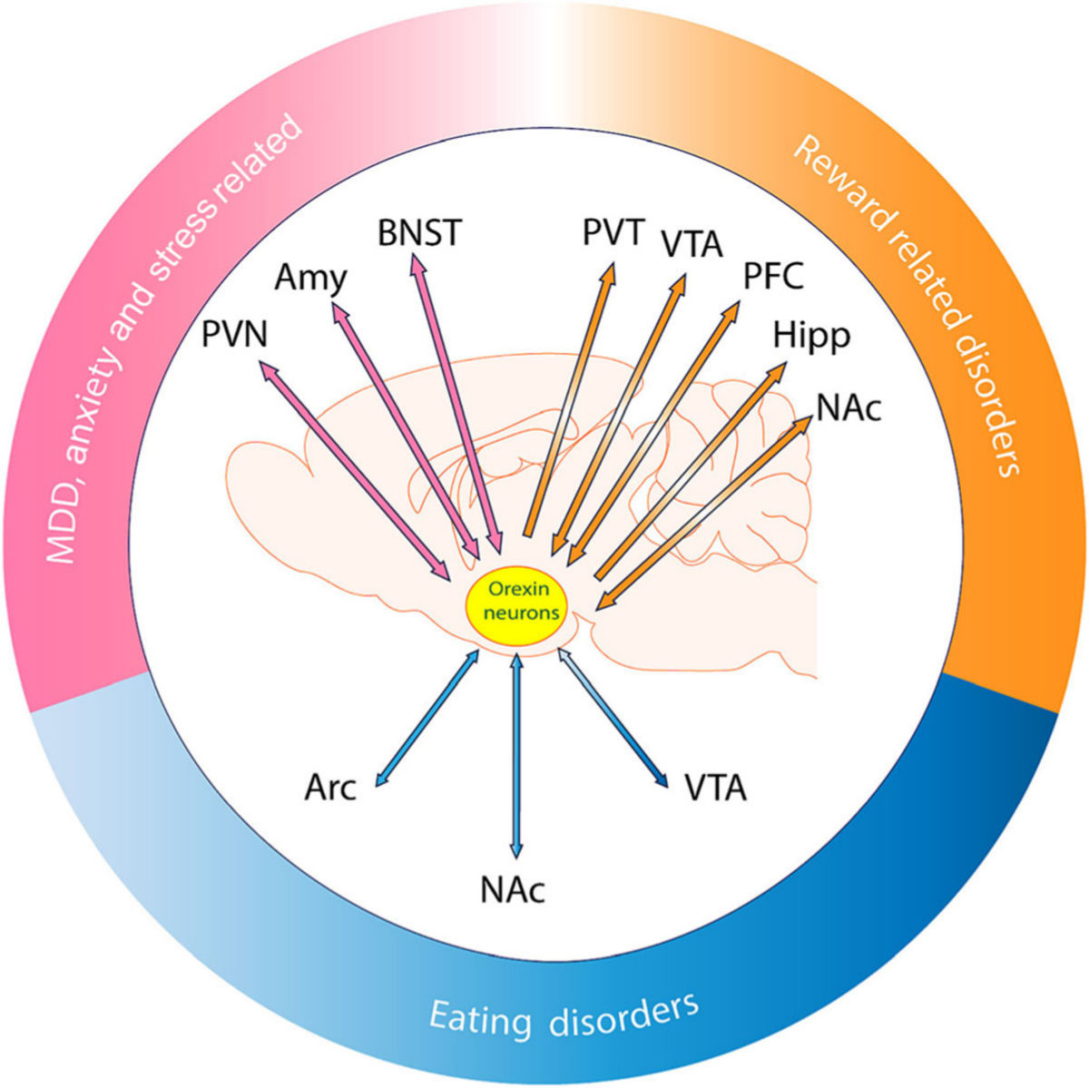
Orexin neurons interconnect with, and integrate input from and output to, brain regions implicated in disorders associated with reward and motivation, eating and metabolism, mood and stress. Abbreviations: Amy, amygdala; Arc, arcuate nucleus; BNST, bed nucleus of the stria terminalis; Hipp, hippocampus; MDD, major depressive disorder; NAc, nucleus accumbens; PFC, prefrontal cortex; PVN, paraventricular hypothalamic nucleus; PVT, paraventricular thalamic nucleus; VTA, ventral tegmental area.

The orexin peptides were initially implicated in feeding (including consummatory actions) and sleep/wake processes. However, later work demonstrated their involvement in a much broader range of physiological processes, from cardiovascular function to cognition (Mahler et al., 2014). Other work, including that by our three independent groups (Christopher V Dayas, Stephanie L. Borgland and Morgan H James), showed that orexins are potent regulators of general reward-directed behaviours, spanning food, drugs and sex, and are also integrally involved in behaviours associated with stress, anxiety and depression (Baimel et al., 2017; Mehr, Mitchison et al., 2021; Yeoh et al., 2014) (Figure 1). How do we reconcile such an apparent diversity of function? One view is that the many behavioural functions of orexin reflect a single unified function in which these peptides translate motivational psychological and physiological need states into directed and energized adaptive behaviours (a role termed ‘motivational activation’) (Mahler et al., 2014). In this framework, orexins coordinate a range of homeostatic functions, including wakefulness, in service of activating adaptive motivational responses to opportunities and/or threats at hand. Consistent with this suggestion, dysfunction of orexin neurons is associated with the manifestation of many behavioural states characterized by aberrant motivation, including eating, substance use, stress and mood disorders.

Central to the role of orexin neurons in coordinating adaptive behaviour is their ability to be recruited by stimuli that demand an energized, motivated response. At a macro level, orexin neurons are most active during waking and the active period, when reward opportunities and threats are most likely, and almost completely inactive during the rest period (Mileykovskiy et al., 2005). Superimposed on periods of high tonic activity (i.e. during the active period) are bursts of phasic activity elicited by physiological and psychological challenges, including hypoxia, food restriction and other stressors, resulting in the recruitment of brain networks necessary to restore homeostasis (Mahler et al., 2014). Critical in the context of this article is evidence that orexin neurons are also acutely activated by environmental stimuli that signal reward availability, such as cues previously associated with drug or food reward, leading to behaviours directed towards reward acquisition (Mahler et al., 2014).

More recently, an unknown mechanism possibly important for how the orexin system functions as a critical regulator of motivated behaviours, is the dynamic regulation of the number of orexin-producing neurons themselves (James & Aston-Jones, 2022). Indeed, the number of immunohistochemically detected orexin neurons in the hypothalamus, including the dorsomedial hypothalamus, perifornical area and lateral hypothalamus (LH) fluctuates across the day-night cycle, peaking during the active period, when neuronal activity is also at its highest (McGregor et al., 2017). This points to a ‘reserve’ population of neurons that can increase orexin peptide production in service of adaptive behaviour (McGregor et al., 2017; James & Aston-Jones, 2022). Indeed, increased orexin gene expression is observed in response to acute challenges, such as caloric restriction, presumably to promote higher orexin peptide availability to invigorate food seeking behaviours (Sakurai et al., 1998). Increasing evidence, however, indicates that this plasticity of peptide production by ‘orexin reserve’ neurons is lost following chronic exposure to highly salient stimuli, resulting in persistently high numbers of orexin neurons. Indeed, the number of orexin-immunoreactive neurons was found to be increased by 54% in the post-mortem brains of persons with opioid use disorder (Thannickal et al., 2018), a phenomenon that is mimicked in laboratory animals across a broad range of species and drugs of abuse (Collier et al., 2019; Collier et al., 2020; Collier et al., 2021; Fragale, James & Aston-Jones, 2021; James et al., 2019; Matzeu & Martin-Fardon, 2021), except for alcohol use disorder, where there is a decrease in the number and size of orexin neurons in mice and humans (McGregor et al., 2023; Olney et al., 2015). Chronic exposure to high fat diets and other palatable foods was also found to increase the total amount of orexin-detectable neurons and orexin mRNA (Mogenson et al., 1983; Wortley et al., 2003). Thus, an emerging view is that disorders of motivation, including substance use and eating disorders, are characterized by a persistent upregulation of orexin peptide production within the reserve neurons, resulting in maladaptive and focused hyper-motivation for food or drug (James & Aston-Jones, 2022).

## 2 | ROLE OF OREXIN IN FEEDING BEHAVIOUR

Feeding behaviour is one typical example of motivated behaviour and is encoded in multiple brain regions. Feeding behaviour is evoked by food-predictive cues as well as by humoral signals that indicate that energy balance is low. In homeostatic feeding, food intake is driven by nutritional need for survival and is governed by metabolic and satiety signals from the gut. The neurocircuits controlling feeding behaviour are thought to be disrupted in pathologies of hypophagia (e.g. resulting in anorexia nervosa) or hyperphagia (e.g. resulting in obesity). In other pathologies, such as substance abuse, the neural circuits traditionally thought to control feeding may be co-opted by drugs of abuse, suggesting overlapping feeding and reward circuitry within the brain (Rossi & Stuber, 2018). A thorough understanding of the mechanisms that regulate food intake is important because of the increasing incidence of eating disorders, as well as obesity. Eating disorders including anorexia nervosa (AN), bulimia nervosa (BN) and binge eating disorder (BED), are among the most complex psychiatric disorders encountered in clinical practice and together contribute significantly to the global burden of disease (Galmiche et al., 2019; Hoek & van Hoeken, 2003).

A role for orexin in feeding was first identified by the Yanagisawa group (Sakurai et al., 1998), who demonstrated that intracerebroventricular (i.c.v.) administration of orexin-A and orexin-B in rats increased consumption of standard chow (Sakurai et al., 1998) and this effect was subsequently confirmed in mice and other species (Haynes et al., 2002; Volkoff et al., 1999; Yokobori et al., 2011). Furthermore, central administration of an orexin antibody or an OX_1_ receptor antagonist, and genetic ablation of orexin cells has been shown to decrease food intake (Hara et al., 2001; Haynes et al., 2000; Yamada et al., 2000). It was originally thought that these effects were secondary to altered wakefulness, especially given that i.c.v. orexin produces long periods of wakefulness and suppresses rapid eye movement (REM) sleep (Mieda et al., 2011). However, the role of orexins in the regulation of feeding is strongly indicated by the existence of reciprocal connections between LH orexin neurons and the arcuate nucleus, a region implicated in homeostatic feeding (Broberger et al., 1998; Elias et al., 1998), and by evidence that Fos immunoreactivity in arcuate neurons is increased following LH infusions of orexin-A (Mullett et al., 2000). Injection of orexin-A into the perifornical area, the rostral portion of LH, the paraventricular nucleus of the hypothalamus (PVN), or nucleus accumbens (NAc), but not in the substantia nigra, increases food intake (Dube et al., 1999; Kotz et al., 2008; Thorpe & Kotz, 2005). Orexin-B increases food intake when injected i.c.v., however the central sites through which these effects are mediated have not been identified (Sweet et al., 1999). One site that showed a very robust increase in food intake was orexin administration in the rostral LH, anterior to the orexin population in the LH (Sweet et al., 1999), and this was enhanced with food restriction (Thorpe et al., 2005). Further, overnight fasting increases excitatory postsynaptic currents onto orexin neurons via promotion of new synapses (Horvath & Gao, 2005), providing a mechanism for this strengthened effect. In addition to increasing food intake, orexin also increases energy expenditure. Given that activation of orexin neuronal projections produces wakefulness and increased activity, an increase in excitatory synaptic input during fasting may also represent a mechanism underlying wakefulness associated with hunger. Notably, following ablation of orexin neurons, mice failed to respond to fasting with increased wakefulness and food-anticipatory activity (Mieda et al., 2004), and are associated with obesity-like phenotypes. For example, these mice became obese despite eating less, because of a lower energy expenditure in the transgenic mice (Hara et al., 2001; Kakizaki et al., 2019). Demonstrating significant role of orexin circuit in maintaining energy homeostasis and that orexin-mediated energy expenditure has a greater impact on body weight than does the effect of orexin-mediated eating behaviour (Kotz, 2006). This is consistent with orexin role in spontaneous physical activity and energy expenditure (Kotz, 2006; Perez-Leighton et al., 2017). Together, orexin has a combined effect of increasing physical activity, which burns calories, and simultaneously increasing feeding behaviour, which increases calories. Thus, orexin neurons are integrating interoceptive signals relayed by the changes in energy state to produce outcomes that serve body homeostasis.

The firing of orexin neurons is directly sensitive to changes in interoceptive signals such as the extracellular concentration of glucose and the appetite regulating hormones ghrelin and leptin (Yamanaka et al., 2003). Leptin and glucose, which typically signal energy replete states, inhibit firing of orexin neurons, while ghrelin, which typically signals hunger, increases firing of orexin neurons (Yamanaka et al., 2003), suggesting orexin neurons are sensitive to energy state. Furthermore, application of 0.2–5 mM glucose induced a postsynaptic membrane hyperpolarization of orexin neurons (Burdakov et al., 2005), whereas application of non-essential amino-acids excited orexin neurons (Karnani et al., 2011). However, orexin neurons may reduce their glucose sensitivity when energy levels are high, reflected by increased lactate, pyruvate or ATP and non-essential dietary amino-acids (Karnani et al., 2011; Venner et al., 2011), suggesting that orexin neurons shift their activity based on metabolic status and are only sensitive to glucose in energy depleted states. Thus, ingested glucose may suppress the orexin-driven net energy expenditure to conserve energy for brain function during severe energy deprivation.

Orexin neuronal activation is also associated with enhanced motivation to work for different kinds of palatable foods (Borgland et al., 2009; Castro et al., 2016; Choi et al., 2010; Kay et al., 2014; Terrill et al., 2016; Thorpe et al., 2005). Microinjection of orexin-A into the nucleus accumbens shell (NAcSh) can increase motivation for food in a progressive ratio task (Choi et al., 2010; Thorpe & Kotz, 2005), in which progressively increased effort is required to obtain rewards. Consistent with this, inhibition of OX_1_ receptor signalling reduces motivation for (Borgland et al., 2009; Nair et al., 2008; Thorpe & Kotz, 2005) and consumption of (Borgland et al., 2009; Choi et al., 2010) food reward in a progressive ratio task, and increases demand elasticity (reduces motivation) for food on a behavioural economics task (Freeman et al., 2021). Notably, these effects appear to be selective for highly palatable food such as high fat food, as the OX_1_ receptor antagonist, SB-334867, did not affect effort for regular food pellets (Hollander et al., 2008). This is not due solely to changes in locomotor activity as 4–20 mg·kg^−1^ SB-334867 doses used in these studies did not influence grooming, sniffing, rearing and locomotion (Rodgers et al., 2001). The role of orexin in motivation for other natural rewards such as sucrose is less clear. For example, SB-334867 did not reduce motivation for sucrose pellets when mice were food restricted (Espana et al., 2010; Rodgers et al., 2001). A recent study suggests that OX_1_ receptor signalling may not be an important driver for sucrose seeking in rats, neither under food-restriction nor food *ad libitum* conditions (Bergamini et al., 2024). In their experimental conditions, neither SB-334867, nor two additional, structurally different OX_1_ receptor antagonist, ACT-335827 and the clinical development candidate nivasorexant (ACT-539313) affected effort-based responding for sucrose in rats. Thus, it is possible that SB-334867 may be acting at OX_2_ receptors to inhibit motivation for food rewards or that inhibition of OX_1_ receptors may induce a selective effect on motivated responding for highly salient reinforcers (addictive drugs, high fat food, but not regular food or sucrose), while leaving more general processes relative to hunger and satiety relatively intact.

Supporting the idea that orexin neurons are associated with consummatory reward, Harris et al. (2005) reported that orexin neurons exhibit robust activation in response to a food-paired context, represented by increased expression of the early intermediate gene, c-*fos* in LH orexin neurons (Harris et al., 2005). As noted above, chronic exposure to a high fat diet can not only increase c-*fos* expression in orexin neurons but also increases the number of orexin expressing neurons in the LH, pointing to a recruitment of an ‘orexin reserve’ population with repeated exposure to highly salient reinforcers (James et al., 2019; James & Aston-Jones, 2022; Thannickal et al., 2018; True et al., 2018). In addition to enhanced motivation, recruitment of these reserve neurons might also mediate symptoms that are often comorbid with disorders of overeating, including anxiety and insomnia (Mehr, Mitchison et al., 2021). Together these studies show that the activation of orexin neurons may act as a critical link between the circuits governing energy balance and those that regulate motivated behaviour.

## 3 | INTERACTIONS BETWEEN THE OREXIN DOPAMINERGIC SYSTEMS IN FEEDING BEHAVIOUR

**FIGURE 2:**
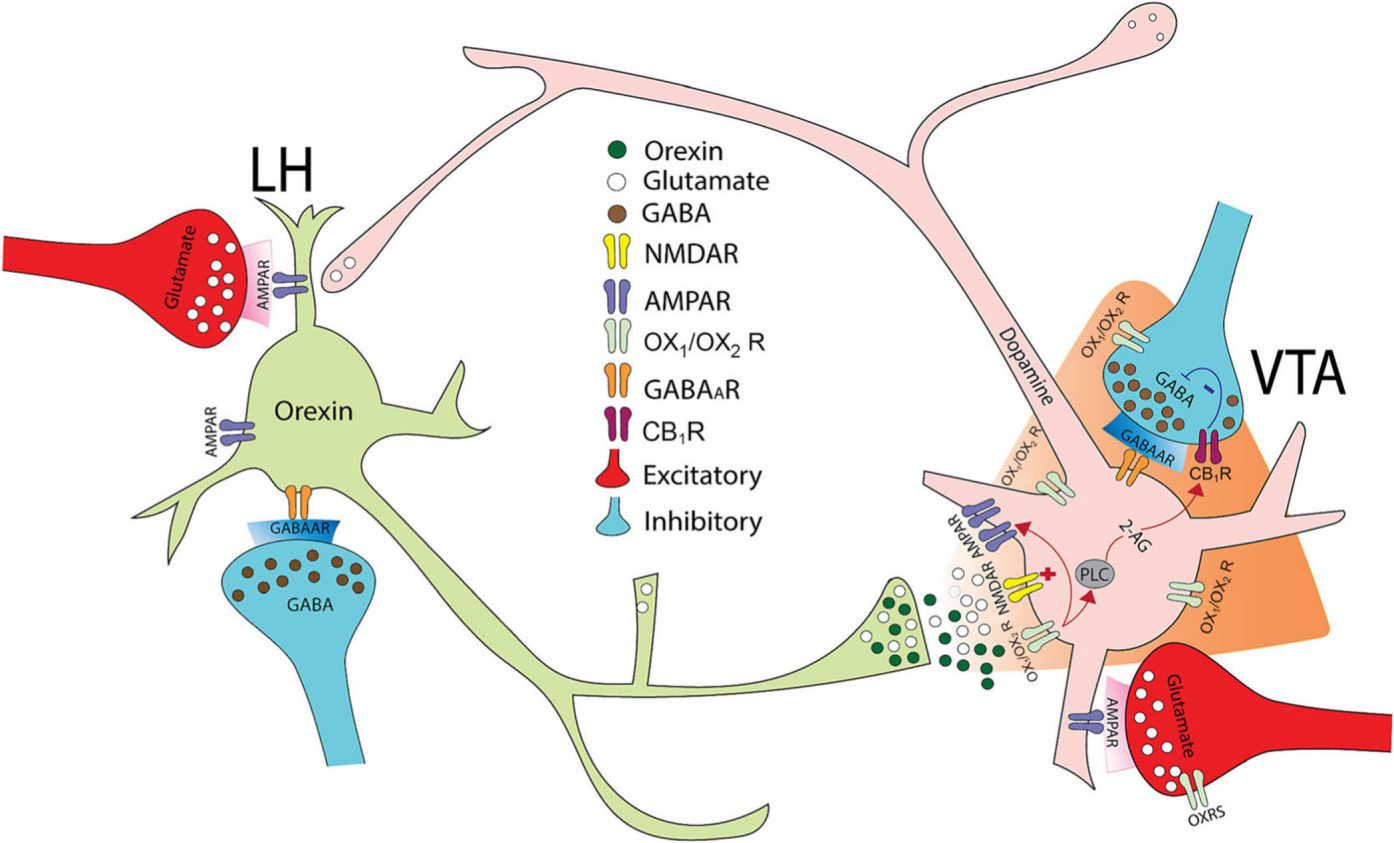
Diagram showing the modulatory function of orexin in the VTA. The VTA is a major target for orexin mediated regulation of reward-seeking behaviours. The modulatory effects of orexin are likely mediated by non-synaptic release of orexin to influence excitatory or inhibitory synaptic transmission.

Two of the LH projections known to be directly relevant to feeding, reward and motivated behaviours include the VTA and NAc. LH orexin-containing neurons send projections that make close apposition with VTA (Baldo et al., 2003; Fadel & Deutch, 2002; Marcus et al., 2001; Peyron et al., 1998; Yoshida et al., 2006) and NAcSh neurons (Lee & Lee, 2016; Liu et al., 2020). In the VTA, orexin axon terminals make synaptic contacts onto dopaminergic and GABAergic neurons, but most are *en passant* fibres projecting to the locus coeruleus (LC) (Balcita-Pedicino & Sesack, 2007; Baldo et al., 2003). Orexin-A increases firing of dopamine as well as GABAergic neurons in the VTA (Baimel et al., 2017; Korotkova et al., 2003). Orexin can induce an endocannabinoid mediated suppression of GABAergic inhibition in the VTA, an effect that disinhibits orexin neurons (Tung et al., 2016). Furthermore, orexin potentiates excitatory synaptic transmission onto dopamine neurons by increasing trafficking of NMDA receptors to the synapse (Borgland et al., 2006; Borgland et al., 2009; Thomas et al., 2022) (Figure 2). Notably, in anaesthetised animals, orexin also potentiates glutamatergic input onto VTA dopamine neurons from prefrontal cortical afferents, further indicating that orexin can amplify concurrent glutamate signalling (Moorman & Aston-Jones, 2010). Orexin microinjected in the VTA can increase accumbal dopamine release (Espana et al., 2011; Vittoz et al., 2008). This is consistent with optogenetic activation of the LH orexin input to the VTA promoting dopamine release under phasic release conditions where glutamatergic afferents to the VTA are likely stimulated (Thomas et al., 2022). Given that activation of dopaminergic neurons increases the incentive motivation for food (Berridge, 2009; Volkow et al., 2017), it is predicted that neuromodulators of dopamine neurons, such as orexin, may drive motivation for food. Using a hedonic feeding model, Terrill et al. (2016) found that VTA administration of orexin-A increased palatable food intake (energy-dense high fat chow) both in rats that had just consumed a large chow meal and in rats given acute daily access to palatable food without a chow preload. Within the same study, intra-VTA orexin-A also increased consumption of a sucrose solution, whereas intra-VTA infusions of the OX_1_ receptor antagonist SB-334867 suppressed sucrose intake, indicating that endogenous OX_1_ receptor stimulation in the VTA plays a role in promoting intake of sucrose under these test conditions. Moreover, orexin potentiation of excitatory synaptic transmission onto dopamine neurons was greater in mice that self-administered highly salient food rewards (Borgland et al., 2009). Intra-VTA blockade of OX_1_ receptors decreased the effort made to obtain palatable food on a barriered T-maze (Thompson & Borgland, 2011) and decreased high fat food intake stimulated by mu (μ) opioid receptor activation in the NAc (Zheng et al., 2007). In one study designed to determine how optical stimulation of orexin in the VTA influences the attribution of incentive value to food cues, mice were trained in a Pavlovian conditioning procedure where orexin inputs to the VTA were stimulated upon delivery of the conditioned stimulus predicting delivery of food pellets. While there was no difference in the value of the cue as lever presses to the cue were unchanged, optogenetic stimulation of orexin inputs to the VTA increased orientation to the food cue (Thomas et al., 2022). Thus, although optogenetic stimulation of orexin in the VTA does not influence the value of the primary reward, it may facilitate the association of cues predicting food. Consistent with this, mice prefer to be in contexts associated with optogenetic stimulation of orexin into the VTA and this preference continues after the optical stimulation, suggesting that mice learn and remember the contextual association (Thomas et al., 2022).

Importantly, orexin is stored in large dense core granular vesicles with dynorphin (Chou et al., 2001; Muschamp et al., 2014) and these neuropeptides have opposing actions on the firing of VTA dopamine neurons (Baimel et al., 2017). It has been proposed that orexin signalling in the VTA can occlude the reward-diminishing effects of dynorphin (Muschamp et al., 2014). OX_1_ receptor antagonism in the VTA decreases cocaine self-administration and LH self-stimulation, both which are reversed by blocking kappa opioid receptors (Muschamp et al., 2014), suggesting that co-released dynorphin is acting as a brake on orexin-mediated reward seeking. Notably, optical stimulation of orexin/dynorphin inputs to the VTA increased dopamine release in the NAc, an effect that was not altered by inhibition of kappa opioid receptors (Thomas et al., 2022). Taken together, cellular actions of orexin-A in the VTA enhances the activity of dopamine neurons, either through increasing firing or excitatory synaptic transmission, leading to increased dopamine release in projection areas of the VTA to drive motivated behaviour and to promote food consumption. These effects may underlie the role of orexin-A in reward-seeking behaviours. These observations provide the main rationale for developing orexin receptor antagonists as a novel and selective approach for treating disorders characterized by excessive motivation, including some eating disorders, because they can reduce activity in the mesolimbic reward system.

### 3.1 | Interactions between orexin and other reward systems in feeding behaviour

Recent discoveries are shedding light on the significance of extrahypothalamic regions in food intake regulation, particularly in the control of cognitive aspects of feeding behaviour related to reward and memory processes (Azevedo et al., 2019; Grill & Hayes, 2012). The hippocampus, a brain region traditionally associated with memory function, has recently emerged as a critical brain region in the control of food intake and feeding-related cognitive processes (see Davidson et al., 2019; Kanoski & Grill, 2017, for review). Suarez et al. (2020) indicated that ghrelin and orexin interact to increase meal size through a descending hippocampus to hindbrain signalling pathway.Specifically, hippocampal ghrelin signalling increased meal size via activation of LH orexin neurons and downstream OX_1_ receptor signalling in the lateral dorsal tegmental nucleus (Suarez et al., 2020). Furthermore, activation of downstream OX_1_ receptors is required for ventral hippocampus ghrelin-mediated hyperphagia, such that ghrelin signalling in the ventral hippocampus engages downstream LH orexin neurons to increase appetite and feeding behaviour (Hsu et al., 2015). Together, these findings reveal novel neurobiological circuitry regulating appetite through which ghrelin signalling in hippocampal neurons engages LH orexin signalling.

Orexin can also directly signal within the hippocampus to produce reward seeking. Orexin can restore potentiation of depressed excitatory synaptic transmission in hippocampal slices (Lu et al., 2016). Microinjection of OX_1_ and OX_2_ receptor antagonists into the dentate gyrus of the hippocampus inhibited the conditioning and expression of morphine (Shirazy et al., 2020) or methamphetamine-induced place preference (Modaberi et al., 2023); currently it is unclear whether these effects generalize to ingestive behaviours.

## 4 | A ROLE FOR OREXIN IN BINGE EATING DISORDER

The obesity epidemic has various proposed causes, one of which is the concept of ‘eating addiction’. This theory posits that certain foods engage similar neural circuits to those recruited by addictive drugs, such that people can exhibit uncontrolled eating patterns that mimic drug-directed behaviours in persons with a substance use disorder (Gearhardt & Hebebrand, 2021). In animal models, binge-like consumption of sugar has been associated with features of addictive behaviour such as escalation of intake, increased anxiety-like behaviour and somatic signs of withdrawal upon cessation of access (Avena, 2010; Brown et al., 2022; Brown & James, 2023).

Binge eating disorder is an eating disorder that was added to the Diagnostic and Statistical Manual of Mental Disorders in 2013 (DSM V., 2013). Binge eating disorder is characterized by regular binge eating episodes during which individuals ingest comparably large amounts of food in a short period of time and experience a perceived loss of control over their eating behaviour (American Psychiatric Association, 2013). It has been proposed that binge eating reflects a pathological compulsion driven by the ‘addictive’ properties of foods. Proponents of this argument highlight the large degree of phenomenological and diagnostic overlap between binge eating disorder and substance use disorders (SUDs), including loss of control over how much is consumed and repeated unsuccessful attempts to abtain from consumption, as well as commonalities in brain structures involved in food and drug craving. Theoretically, addiction and traditional eating disorder perspectives offer distinct explanations for loss of control overeating. The ‘food addiction’ viewpoint emphasizes the addictive nature of highly processed foods, suggesting they can hijack the reward system in susceptible individuals. In contrast, traditional eating disorder models focus on factors like rigid dietary restraint, shape and weight concerns. Despite differences, both perspectives recognize common contributors such as impulsivity, reward dysfunction and emotion dysregulation to eating psychopathology (Fletcher & Kenny, 2018; Gearhardt et al., 2014). Notably, several groups consider the concept of food addiction highly contentious because of its supposed oversimplification of complex behavioural phenomena and the possible stigma associated with this terminology (Finlayson, 2017; Fletcher & Kenny, 2018).

A substantial body of evidence now indicates that orexin influences overeating primarily via actions at OX_1_ receptors. Systemic administration of OX_1_ receptor antagonists reduces binge-like consumption of a range of palatable diets in rats and mice (Alcaraz-Iborra et al., 2014; Olney et al., 2015;Piccoli et al., 2012; Rorabaugh et al., 2014; Vickers et al., 2015). Both selective OX_1_ receptor and dual OX_1_/OX_2_ receptors antagonists (DORAs), but not selective OX_2_ receptor antagonists reduce binge-like eating, often at doses that do not affect homeostatic feeding (Piccoli et al., 2012; Rorabaugh et al., 2014; Vickers et al., 2015). Injection of orexin-A into the third cerebral ventricle selectively increased a preference for high-fat diet (Clegg et al., 2002), whereas SB-334867, a selective OX_1_ receptor antagonist, blocked orexin-A-induced orexigenic effects and induced anorectic and anti-obesity activities in obese animals (Rodgers et al., 2001). Peripheral administration of SB-334867 was also reduced the intake of palatable food (Choi et al., 2010) and attenuated yohimbine-induced reinstatement of extinguished sucrose seeking (Richards et al., 2008). In general, blockade of OX_1_ receptors signalling is highly effective at reducing binge-like and other forms of motivated intake of a range of palatable foods, including chocolate, sucrose, saccharine and fructose, and these compounds are also effective at suppressing cue-induced reinstatement of extinguished food seeking (Alcaraz-Iborra et al., 2014; Castro et al., 2016; Piccoli et al., 2012; Rorabaugh et al., 2014). Taken together, there is strong evidence for a role of orexin signalling at the OX_1_ receptor in binge eating in rodent models. Notably however, a recent study reported that a novel, highly selective OX_1_ receptor antagonist (nivasorexant) failed to reduce progressive ratio responding for sucrose pellets. Interestingly, there was a trend towards an effect at a high dose of nivasorexant that begins to engage the OX_2_ receptor, indicating a potential role for OX_2_ receptor in these processes (Bergamini et al., 2024).

Counterintuitively, people with narcolepsy, a deficiency of orexin signalling, have increased prevalence of eating disorders (Fortuyn et al., 2008), including binge eating disorder (Dimitrova et al., 2011). Notably those with narcolepsy also have higher scores on impulsivity scales and impulsivity is strongly associated with binge eating disorder (Dimitrova et al., 2011), and thus, binge eating disorder in narcolepsy may be driven by impulsive behaviours. Alternatively, it is possible that the loss of orexin neurons having profound implications on maintaining wakefulness can influence food intake to maintain arousal states, an effect that could potentially lead to binge episodes. Notably, a placebo-controlled, double-blind phase 2 clinical study by McElroy et al., 2023, indicated that a well-tolerated OX_1_ receptor antagonist, ACT-589313 (nivasorexant) did not show improvement over placebo in reducing binge eating days per week in 136 adult patients with binge eating disorder (McElroy et al., 2023). In conclusion, given the strong evidence in rodent models, further studies examining the role of orexin receptor signalling in obesity and binge eating disorder in humans without narcolepsy are required. While targeting the orexin system holds promise in advancing our understanding of feeding disorders, the complexity of overeating pathology suggests that more preclinical and clinical studies are clearly needed this may include what binge eating disorder endophenotypes are sensitive to orexin modulation.

## 5 | ROLE OF OREXIN IN DRUG-SEEKING AND ADDICTION

A general role for orexin neurons in drug reward was first shown by Harris et al. (2005), who reported that orexin neurons are recruited in rats exhibiting a conditioned place preference (CPP) for morphine or cocaine. Subsequent work has since demonstrated that orexin neurons are recruited by discriminative stimuli associated with almost all drugs of abuse, including cocaine, morphine, ethanol and nicotine, and that in many cases, the magnitude with which orexin neurons are activated corresponds to drug seeking behaviours are responsive to environmental cues associated with morphine, ethanol and cocaine (Dayas et al., 2008; Harris et al., 2005; Martin-Fardon et al., 2010; Martin-Fardon et al., 2018; Pasumarthi et al., 2006). Moreover, rats with higher numbers of orexin immunoreactive neurons exhibit higher motivation for cocaine Pantazis et al., 2019. Consistent with this, inhibition of orexin signalling (Narita et al., 2006) or orexin peptide knockout mice have reduced opioid seeking behaviours (Narita et al., 2006; Giannotti et al., 2022) and attenuated morphine withdrawal (Georgescu et al., 2003). Importantly however, not all studies have shown that the specific recruitment of LH orexin neurons is necessary for the renewal of drug seeking by drug-associated contexts. For example, Marchant et al. (2009) showed that LH cell recruitment is increased under conditions of both test and the context specifically associated with beer and sucrose renewal. Further, microinjection of an antisense morpholino oligomer into the LH prevented renewal of alcohol-seeking, at a concentration that reduced both melanin-concentrating hormone (MCH) and orexin protein levels in the LH (Prasad & McNally, 2014).

Orexin signalling has been implicated in binge-like ethanol consumption. Systemic administration of OX_1_ or OX_2_ receptor antagonists can block binge-like consumption of alcohol as well as sucrose (Anderson et al., 2014; Moorman et al., 2017). However, high-effort self-administration for alcohol, but not sucrose, is reduced following SB-334867 (Jupp et al., 2011). Studies aimed at identifying the sites of action responsible for orexin-mediated effects on drug-seeking have focused on the paraventricular thalamus (PVT), prefrontal cortex, hippocampus, central amygdala (CeA) and VTA. Injections of the OX_1_ receptor antagonist, SB-334867, into the VTA suppressed discriminative and discrete cue-induced cocaine-seeking behaviour in rats (James et al., 2011; Mahler & Aston-Jones, 2012; Pantazis et al., 2022). Interestingly, while one study reported that SB-334867 injections into the PVT had no effect on cue-elicited cocaine-seeking, a separate study showed that injections of the orexin-A peptide into the posterior PVT was sufficient to reinstate cocaine seeking (Matzeu et al., 2016) in rats with a history of extended (6 h·day^−1^) cocaine self-administration. This priming effect of orexin-A was blocked by co-administration of the OX_2_ receptor antagonist, TCS-OX2-29, but not the OX_1_ receptor antagonist SB-334867, indicating an important role for OX_2_ receptor signalling in the posterior PVT. Barson et al. (2015) found that ethanol consuming rats exhibited increased orexin mRNA levels in the hypothalamus and upregulation of the OX_2_ receptor, while the OX_1_ receptor remained unchanged (Barson et al., 2015). These effects were observed specifically in the anterior PVT, with no significant changes in the posterior PVT. Similarly, ethanol gavage resulted in elevated double labelling of c-Fos with the OX_2_ receptor, but not the OX_1_ receptor, particularly in the anterior PVT. Administration of orexin-A or orexin-B in the anterior PVT, but not the posterior PVT augmented ethanol consumption. Ethanol intake was reduced by anterior PVT administration of the OX_2_ receptor antagonist, TCS-OX2-29, while OX_1_ receptor antagonist SB-334867, had no significant effect (Barson et al., 2015). Olney and colleagues identified potential downstream brain regions in which orexin exerts its effects on alcohol seeking. Using region-directed infusion of SB-334867 within the VTA or central amygdala, the authors demonstrated that OX_1_ receptor is the predominate receptor subtype within these two regions that regulates binge-like ethanol drinking. Interestingly, inhibition of OX_1_ receptor within the VTA and central amygdala did not affect binge-like sucrose intake, showing that natural rewards are likely regulated via different neuronal circuits (Olney et al., 2017). Similarly, Brown and colleagues used a cue-induced model of reinstatement for ethanol in alcohol-preferring rats and found that prelimbic prefrontal cortex or VTA-specific infusions of SB-334867 attenuated reinstatement (Brown et al., 2016).

The identification of the VTA as a site responsible for the reinstating actions of orexins is consistent with work demonstrating that intra-VTA orexin increased the firing rate of VTA dopamine neurons (Korotkova et al., 2003; Muschamp et al., 2014) and increases extracellular dopamine in other reward-related brain regions such as the NAcSh and the prefrontal cortex (Narita et al., 2006; Vittoz et al., 2008). Administration of the orexin antagonist SB-334867 into VTA can reduce preference for a morphine-paired environment in rats (Narita et al., 2006). Similarly, orexin-A infusion into VTA reinstated conditioned place preference for the morphine paired environment (Harris et al., 2005). Tung et al. (2016) showed that orexin neurons contribute to stress-induced cocaine relapse by actions at VTA dopamine neurons. Additionally, they showed that activation of postsynaptic Gq-coupled orexin receptors on VTA dopamine neuron increased retrograde endocannabinoid signalling at presynaptic GABA inputs to suppress GABA release and shift the excitatory balance of VTA dopamine neurons (Tung et al., 2016). As such, intra-VTA orexin-induced reinstatement of extinguished cocaine CPP was blocked in cannabinoid 1 (CB_1_) receptor knockout mice or in the presence of a CB_1_ receptor antagonist.

In addition to VTA, a number of other cortico-striatal brain regions are thought to interface with the orexin system to mediate cue-induced drug behaviours. A functional NAc-LH pathway has been implicated in context-induced reinstatement (renewal) of reward seeking and extinction (Marchant et al., 2009). Local infusions of SB-334867 or TCS-29-029 into the NAcSh prevent stress-induced reinstatement of morphine conditioned place preference (Qi et al., 2013) and injections of TCS-OX2-029 in NAc reduced responding for ethanol (Brown et al., 2013). Inhibiting OX_1_ receptors in NAcSh or the ventromedial prefrontal cortex is effective in reducing alcohol binging in adult male C57BL6/J mice (Lei et al., 2016; Lei et al., 2019). In contrast, OX_2_ receptor inhibition in NAcSh does not alter alcohol drinking and OX_1_ receptor inhibition in NAcSh does not reduce saccharin intake (Lei et al., 2016). In addition, inhibiting NAcSh OX_1_ receptors significantly decreases alcohol intake in mice under two different bottle-based alcohol drinking paradigms, a limited daily access model (2 h per day) and on an intermittent access model (three overnight alcohol access sessions per week, with at least 24 h per drinking period) (Lei et al., 2016; Lei et al., 2019). Importantly, inhibiting NAcSh OX_1_ receptors is particularly effective at reducing the higher alcohol intake levels in excessively-drinking mice, with no average effect in more moderate bingers (Lei et al., 2019). Their results strongly suggest that the NAcSh is a critical region where OX_1_ receptor activation promotes excessive alcohol intake in higher drinking individuals, providing the first link between a specific brain signal (NAcSh OX_1_ receptors) and orexin regulation of intake in excessive drinkers (see Hopf, 2020, for review).

The dorsolateral prefrontal cortex displays increased activation in response to drug cues in subjects experiencing alcohol-dependence (George et al., 2001). An anatomical connection between orexin neurons and the prefrontal cortex exists, and there is dense OX_1_ receptor expression in the prefrontal cortex (Nambu et al., 1999). Further, prefrontal cortex pyramidal cells are directly excited by orexin-A innervation (Xia et al., 2005). Administration of orexin into the orbitofrontal cortex or insula increased Fos activation, food intake, as well as an enhances an orofacial licking response to sucrose associated with a hedonic ‘liking’ reaction (Castro & Berridge, 2017). Intra medial prefrontal cortex administration of SB-334867 attenuated the acquisition and expression of CPP for morphine or food (Jamali et al., 2021). Brain regions where SB-334867 modulates relapsed alcohol-seeking shifts from the orbitofrontal/prelimbic cortex and NAc core following immediate reinstatement to primarily a cortical locus following delayed reinstatement, showing that the effects of orexin involvement maybe both brain region and time-dependent (Jupp et al., 2011).

Orexin-induced drug-seeking behaviour can be blocked by adrenoceptor and CRF receptor antagonists, thus highlighting an important role of orexin in coordinating motivated behaviours in response to stress (Boutrel et al., 2005). The pharmacological stressor yohimbine can produce reinstatement to alcohol seeking in rats by enhancing CRF in the central amygdala (Funk et al., 2006), this effect is blocked by administration of the OX_1_ receptor antagonist SB-334867, suggesting that inhibition of OX_1_ receptors can prevent the CRF-mediated stress response in yohimbine-induced alcohol reinstatement (Richards et al., 2008). There also exists a functional connection between LH orexin neurons and the brainstem, with orexin neurons directly innervating relaxin-3 neurons of the nucleus incertus. In alcohol preferring rats, stress-induced reinstatement of alcohol seeking can be blocked using the OX_2_ antagonist, TCS-OX2-29, administered into the nucleus incertus. Infusions of the OX_1_ antagonist, SB-334867, in the same region did not affect reinstatement (Kastman et al., 2016). An interesting study by Ubaldi et al. (2016), provided evidence for a relationship between orexin and neuropeptide S (NPS) system from within the pericoerulear zone of the brainstem. Microinjection of NPS into the LH facilitated discriminative cue-induced reinstatement of alcohol seeking, an effect that was attenuated by SB-334867 microinjection into PVN or bed nucleus of the stria terminalis (BNST), but not the VTA (Ubaldi et al., 2016), suggesting that reinstatement of cue-induced alcohol seeking is also regulated by stress-sensitive brain regions, including the PVN and the bed nucleus of the stria terminalis. Furthermore, in rats exposed to long access (6 h) cocaine self-administration, intracentral amygdala infusion of SB-334867 blocked stress-induced reinstatement of cocaine-seeking (Schmeichel et al., 2017). Electrophysiological recordings from this study suggested that stress-induced reinstatement of cocaine seeking may be modulated via GABAergic transmission within the central amygdala. Thus, orexin signalling in stress-sensitive brain regions plays a role in stress- or cue-induced reinstatement of cocaine or alcohol seeking.

Chronic drug use can cause long lasting anatomical and functional alterations in orexin neuron circuitry. Recent work showed that prolonged cocaine consumption can increase the number of orexin neurons in the hypothalamus of rodents, observed in two different models of addiction-like behaviour (James et al., 2019; Matzeu & Martin-Fardon, 2021). This effect extended from 2–3 weeks and up to 150 days following the last cocaine exposure and was causally linked with cocaine motivation (demand elasticity) using a behavioural economics task. Similarly, a fixed dose (100 mg·kg^−1^) or an escalating dose (10–180 mg·kg^−1^) of morphine administered for 14 days (but not 7 days), led to an increase in the number of orexin-producing cells in wild-type mice, but with a decrease in their cell body size (Thannickal et al., 2018). This increase in orexin cell count was not attributed to neurogenesis and persisted for several weeks after the cessation of morphine treatment. The number of melanin-concentrating hormone neurons, located in the same hypothalamic region as orexin-producing cells, remained unchanged following morphine administration (Thannickal et al., 2018). They also found that in post-mortem LH samples of opioid users there was a 50% increase in the number of orexin neurons compared to other non-opioid user samples (Thannickal et al., 2018). However, this contrasts with humans with alcohol use disorder who have a reduced number and size of detectible orexin and melanin-concentrating hormone neurons (McGregor et al., 2023). A recent study in newborn rats demonstrates that embryonic ethanol exposure at low-moderate doses increases the density as well as proliferation and differentiation of orexin and melanin-concentrating hormone neurons in the LH where they are normally concentrated. In addition, they provide new evidence that ethanol induces ectopic orexin and melanin-concentrating hormone neurons outside the hypothalamus in more anterior regions in the nucleus accumbens core and ventromedial caudate putamen where they have not been previously observed (Collier et al., 2022). Thus, prenatal alcohol exposure may induce epigenetic changes to promotor regions of the orexin-A (hypocretin-1 [*Hcrt*)]) gene.

This follows earlier work that explored how addictive drugs might modify the functional properties of LH orexin neurons. For example, the LH was found to display the most significant pre and post synaptic gene expression alterations in response to cocaine intake compared to other brain regions examined including the NAc, prefrontal cortex and VTA (Ahmed et al., 2005). Other research suggests that the LH-orexin circuitry can adapt rapidly to changes in metabolic and environmental conditions such as food restriction and sleep deprivation (Horvath & Gao, 2005; Rao et al., 2007). Yeoh et al. (2012) expanded on these findings to show that cocaine exposure induces plasticity in orexin and non-orexin neurons in the LH. Specifically, in both rats and mice, withdrawal from cocaine increased pre-synaptic excitatory drive onto orexin neurons, regardless of whether cocaine was self-administered or experimenter administered. But, whereas acute withdrawal from cocaine only increased pre-synaptic drive to orexin neurons, at more protracted time points (e.g. 2 weeks after the last cocaine injection), cocaine also promoted post-synaptic adaptations, increasing the AMPA:NMDA ratio in orexin neurons (Yeoh et al., 2019). Thus, it appears that the increased glutamate drive that occurs during drug exposure results in increased post-synaptic strength following more protracted withdrawal. To determine whether normalizing cocaine-induced glutamate release might be relevant to treating drug-relapse, the same study targeted group III metabotropic glutamate (mGlu_4,6,7,8_) receptors, which are highly expressed in lateral hypothalamus. Injections of the group III mGlu agonist L-2-amino-4-phosphonobutyric acid (L-AP4) into LH prevented discriminative cue-elicited drug seeking following a period of extended (14 days) abstinence. Interestingly, given the evidence presented above regarding the impact of food deprivation on excitatory drive to orexin neurons, L-AP4 injections also suppressed Fos in LH-orexin elicited by food restriction, indicating functional inhibition of these neurons. Notably, injections of the mGlu receptor agonist had no impact on locomotor activity nor lever pressing for food in sated animals, indicating specificity of behavioural effects to drug-seeking outcomes (Yeoh et al., 2019). L-AP4 treatment also increased centre square time in the open field assay, perhaps indicating that its effects on drug seeking might be related to anxiolytic effects of reducing orexin cell activity. This interpretation aligns with a body of evidence linking orexins with mood and anxiety behaviour, discussed in further detail below. We suggest that LH orexin neurons are involved in pathological drug-seeking processes, but the involvement of this system is likely important to energize motivated behaviour necessary for reward-seeking through processes similar to that described above for food.

## 6 | OREXIN NEURONS ARE RESPONSIVE TO STRESS AND AROUSAL

Behaviours modulated by orexins are commonly dysregulated in disorders of mood and emotion, including sleep/arousal, reward sensitivity, cognition and stress responsiveness (Baimel et al., 2015; Giardino & de Lecea, 2014; Nevárez & de Lecea, 2018; Villano et al., 2017). Accordingly, it is not surprising that alterations in orexin circuitry are associated with neuropsychiatric conditions. In animal models, acute stress, including the forced swim test, restraint stress and the stress of intraperitoneal injections are associated with an upregulation of orexin activity (Chang et al., 2007; Grafe et al., 2017; Panhelainen & Korpi, 2012) while chronic stress-induced depression-like behaviour using a rat social defeat model decreases basal orexin activity (Nocjar et al., 2012). James et al. (2014) found that maternal separation, a model of early life stress (ELS) in rats, impaired stress-induced activation of orexin neurons in adulthood, which was associated with reduced exploratory activity in the open field. Interestingly, free access to running wheels during postnatal day 40–70 in male rats reversed these effects in males, but exacerbated them in females, indicating a sex-dependent role for this type of exercise on orexin system function. Work from Yaeger et al., 2022 demonstrates that OX_1_ receptors in the basolateral amygdala (BLA) have an important role in stress-related behaviours. In this study (Yaeger et al., 2022), the authors show that acute pharmacological inhibition (SB-674042) of OX_1_ receptors in the basolateral amygdala promotes behavioural shifts from stress sensitive to stress-resilient responses in mice that undergo a social stress paradigm. This suggests that orexin receptors can coordinate balance between pro- and anti-stress responses within the basolateral amygdala. Additionally, orexin signalling may underlie the antidepressant-like effect of calorie restriction (Lutter et al., 2008). They demonstrate that 10 days of calorie restriction, corresponding to a 20%–25% weight loss, causes a marked antidepressant-like response in two rodent models of depression and that this response was dependent on orexin signalling (Lutter et al., 2008).

Orexins mediate mood and stress outcomes, at least in part, via their interaction with the hypothalamus-pituitary–adrenal (HPA) axis. Orexin neurons make functional connections with PVN neurons that release corticotropin-releasing hormone (CRF), the group of neurons that form the apex of the HPA axis. Using immunohistochemical techniques, Winskey-Sommerer et al. (Winsky-Sommerer et al., 2004) demonstrated that orexin neurons receive synaptic input from CRF boutons, while CRF_1_ and CRF_2_ receptors are commonly present on orexin-positive neurons. Further, bath application of CRF to orexin-containing brain slices dose-dependently depolarizes membrane potential and increases firing rate of a subpopulation of orexin neurons. The CRF-induced depolarisation of orexin neurons was inhibited by slice pre-treatment with the peptidergic CRF_1_ receptor antagonist astressin, an effect that was absent with pre-treatment of CRF_2_ receptor selective agonist urocortin 3 (stresscopin; Winsky-Sommerer et al., 2004). Importantly, the PVN-orexin relationship is bidirectional, as electrophysiological bath application of orexin-B elicits excitatory effects on PVN neurons (Shirasaka et al., 2001). In support, there is abundant expression of OX_2_ receptors expressed on PVN neurons (Marcus et al., 2001), and selective photo-stimulation of orexin neurons increases output of HPA axis activity, as measured by plasma corticosterone (Bonnavion et al., 2015). From clinical studies, patients exhibiting panic-related anxiety present with elevated orexin-A expression in the LH (Johnson et al., 2010) or in the cerebrospinal fluid (CSF) of rodents (Grafe et al., 2017) or humans (Johnson et al., 2010). Interestingly, in patients that had attempted suicide, orexin-A expression in the CSF was correlated positively with CSF levels of the stress hormone CRF (Brundin et al., 2007). This is consistent with another study demonstrating increased CSF orexin levels 1 year after the suicide attempt (Brundin et al., 2009). However, in suicidal patients with major depressive disorder (MDD), there was a reduction in orexin in the CSF associated with a loss of circadian rhythm (Brundin et al., 2007), although other studies have demonstrated no change in CSF levels in humans with with major depressive disorder (Schmidt et al., 2011). Overall, these data suggest that bidirectional changes in orexin release can lead to different behavioural outcomes and neuropsychiatric symptomatology.

Persistent changes in the activity of the HPA axis is a feature of hyper arousal and chronic stress. Similar observations have been observed in animal models. For example, using the unpredictable chronic stress (UCS) paradigm rodents displayed plasma corticosterone increase and adrenal gland hypertrophy (Ulrich-Lai et al., 2006). A study using mild unpredictable chronic stress found that mice had elevated orexin neuron activity in the dorsomedial/perifornical hypothalamic area, but not the lateral hypothalamic area, as measured using Fos protein. Interestingly, chronic injection of fluoxetine, an SSRI antidepressant, reversed the unpredictable chronic stress -induced orexin hyperactivation (Nollet et al., 2011). A follow-up study used almorexant, the dual orexin receptor antagonist, which produced antidepressant-like effects following mild unpredictable chronic stress and normalized hyperactive HPA axis activity following stress (Nollet et al., 2012). Similarly, OX_1_ receptor knockout mice displayed a decrease in depressive-like behaviour in the forced swim test and tail suspension test (Scott et al., 2011). Orexin levels in the CSF are increased with acute stress, but significantly reduced with 5 days of repeated restraint stress (Grafe et al., 2017). Designer receptors exclusively activated by designer drugs (DREADDs) activation of orexin release was also suppressed with HPA activation by 5 days of restraint stress. While the OX_2_ receptor antagonist blocked the acute stress response and also, reduced elevated plasma ACTH response to acute and repeated stress under conditions of high orexin release (Grafe et al., 2017). Overall, these studies highlight the importance of orexin hyperactivity in the development of stress-induced behaviours relevant to depressive disorders.

Common features of depression include changes to measures of arousal, food and reward-seeking and other motivational disturbances, functions that are modulated in part by the orexin system. Work from Campbell et al. (2017) demonstrated that early life stress (ELS) impaired motivation for sucrose self-administration under progressive ratio conditions. Recovery of this depressive-like behavioural state was achieved by using a chemogenetic approach in which excitatory-DREADDs were expressed in the LH. While this approach used a panneuronal promoter and thus did not target any specific cell type in the LH, the DREADD agonist clozapine-N-oxide did increase the activity of putative-orexin neurons in slice electrophysiology experiments and Fos activity was increased in the LH orexin neurons but not dorsomedial hypothalamus (Campbell et al., 2017). Thus, disruption of orexin signalling may contribute to the motivational impairments observed in this study (Grafe & Bhatnagar, 2018).

## 7 | POTENTIAL INPUTS ONTO OREXIN RELEVANT TO REWARD AND STRESS BEHAVIOURS

In general, there has been much greater investigation of the projection targets of orexin neurons compared to their inputs. Sakurai et al. (2005) used a viral-mediated tetanus toxin approach to map afferent synaptic inputs to orexin neurons. They discovered that orexin neurons receive input from several neuronal areas involved in wakefulness, including serotonergic neurons in the raphe nucleus and cholinergic neurons from the basal forebrain. Interestingly, areas known to be involved in emotional or stress regulation also send projections to orexin cells, including the amygdala, the bed nucleus of the stria terminalis, NAcSh and the hypothalamus. More recently, Cre-dependent expression of the retrograde pseudorabies virus highlighted a similar pattern of connectivity onto orexin neurons, including striatal and hypothalamic inputs (Giardino et al., 2018; González et al., 2016). Using doubled-labelled fluorescent and monosynaptic retrograde tracing approaches, Saito et al. (2018) found that PVN CRF neurons send direct projections onto orexin neurons. An intriguing finding from two of these studies observed that neurons within the LH (presumably from both GABAergic (Ferrari et al., 2018) and glutamatergic (Li et al., 2002) populations) send the strongest direct projections to orexin neurons (Giardino et al., 2018).

In addition, early studies using electrophysiological recordings and retrograde tracings identified an inhibitory pathway from the NAcSh to the LH (Kirouac & Ganguly, 1995; Mogenson et al., 1983). Within the NAcSh there are two populations of dopamine receptor-expressing neurons, dopamine 1 (D_1_) receptor and 2 (D_2_) (Bertran-Gonzalez et al., 2008). Pharmacological inhibition of the NAcSh triggers intense feeding and an upregulation of Fos protein in LH neurons (Baldo et al., 2004; Reynolds & Berridge, 2001; Stratford & Kelley, 1999). Baldo et al. (2004) found that infusion of muscimol into the shell activates orexin-labelled LH cells, but not melanin-concentrating hormone-labelled cells and this treatment also induces strong Fos expression in the arcuate nucleus (Baldo et al., 2004). The authors proposed that the NAcSh-LH pathway serves as a crucial communication route between striatal and hypothalamic mechanisms controlling motivated behaviour (Kelley, 1999). These findings suggest that the medium spiny neurons within the NAcSh, which contain GABA as their major transmitter, may release GABA in association with normal feeding, which by self-inhibition (through recurrent collaterals) would result in disinhibition of LH orexin neurons or perhaps other downstream cells involved in feeding. Moreover, they suggest that converging corticolimbic input to the NAcSh, coded by glutamate, restricts this mechanism, and exerts inhibitory control over downstream feeding circuits. This mechanism may be necessary to override strong, metabolically driven feeding signals when an animal, engaged in feeding, directs its attention to novel or potentially dangerous signals in the environment. Thus, the NAcSh acts as a kind of sensory ‘sentinel’, enabling adaptive switching and shutting off downstream feeding motor pattern generators, via the LH (Kelley et al., 2005). An interesting study by O’Connor et al. (2015) demonstrated that optogenetic stimulation of NAcSh terminals in the LH produced inhibitory postsynaptic currents on LH GABA neurons. Using D_1_ and D_2_ receptor transgenic mice and viral-assisted circuit mapping, the majority of inhibitory projections from the NAcSh originated from D_1_ neurons. Notably, photo-stimulation of these terminals in the LH caused rapid termination of consummatory behaviour. Interestingly, post-hoc labelling using neurobiotin suggested that the identity of the LH neurons receiving NAcSh input are orexin-negative, most likely GABAergic (Yeoh et al., 2012). Thus, further work is required to understand ventrostriatal inputs that modulate LH orexin cell responses given the important work by several groups defining the roles of LH GABA and glutamate cell types in consummatory, reward and aversive behaviours, respectively (Jennings et al., 2013; Stuber & Wise, 2016).

As discussed above, orexin neurons receive synaptic connections from CRF neurons with the PVN, critical for regulating stress responses. Mitchell et al. (2024) demonstrated that repeated optogenetic stimulation of the PVN CRF inputs to the LH increased acute stress-related behaviours and produced long-lasting deficits in the motivational drive for sucrose. This was associated with increased Fos-protein expression in the LH. Direct stimulation of the PVN CRF inputs in the LH produced a similar pattern of motivational impairments. This work is interesting in the context of PVN’s previously known roles in both stress and reward. For example, Yaun and colleagues’ work using fibre photometry in mice demonstrated that sucrose reward inhibits stress-induced burst firing in PVN CRF neurons as well as modulating the behavioural and hormonal responses following stress (Yuan et al., 2019). Future research should explore the complex interplay between PVN CRF and orexin in regard to stress and reward sensitivity. Considering the PVN and associated stress responses also have known roles in drug seeking (Barson et al., 2010) and regulation of food intake (Hill, 2012), this suggests PVN-orexin circuitry coordinates multiple complex behaviours.

## 8 | THERAPEUTIC INHIBITION OF OREXIN SIGNALLING FOR PSYCHIATRIC DISORDERS

Modulation of orexin neuron circuitries through novel pharmacological approaches could assist in modulating neuropsychiatric pathologies characterized by aberrant motivation, including substance use and mood disorders (James et al., 2020; James & Aston-Jones, 2020; James et al., 2021; Fragale, James, Avila, et al., 2021; Mehr, Bilotti et al., 2021; Zhang et al., 2024). Suvorexant is a dual-orexin receptor antagonist (DORA) that is effective in improving sleep duration and continuity (Herring et al., 2012; Herring et al., 2016). Suvorexant has recently garnered attention as a potential strategy for normalizing sleep disturbances during abstinence in patients with opioid use disorder (OUD). In a randomized, double-blind, placebo-controlled clinical trial in which individuals undergoing a stepwise buprenorphine/naloxone taper, opioid use disorder patients were administered either 20 or 40 mg of suvorexant or a placebo. Suvorexant treatment was associated with clinically relevant enhancements in sleep duration, reductions in opioid withdrawal symptom severity post-taper and decreases in various dimensions of opioid craving throughout the study. This indicates that suvorexant may have therapeutic potential in mitigating the negative impact of opioid withdrawal on sleep quality. (Huhn et al., 2022). Although the exact mechanisms mediating these effects are unclear, it is possible that suvorexant might reduce drug craving by (1), a top down mechanism, whereby improved sleep improves next-day executive function and (2), a bottom-up mechanism, in which orexin receptor blockade directly reduces craving (Gyawali & James, 2023). Ongoing clinical trials are also addressing the efficacy of suvorexant in sleep efficiency and opioid abstinence in outpatient groups (NCT04262193), as an adjuvant to buprenorphine treatment in fentanyl users (NCT05145764) and the effects of sleep and stress in early recovery from opioid use disorder (NCT04287062). Furthermore, suvorexant is under investigation for use in alcohol use disorder (NCT05656534 and NCT04229095), nicotine addiction (NCT04234997) and post-traumatic stress disorder (NCT03642028). Furthermore, the OX_2_ receptor antagonist, seltorexant, is currently being examined as an adjunctive therapy in adults and elderly participants with major depressive disorder with insomnia symptoms (NCT04533529). Importantly, targeting the orexin system for therapeutic gain will require a thorough understanding of the complex interactions of this system to pave the way to develop novel therapeutic strategies for disorders involving orexin dysfunction. Treatment of specific orexin dependent behavioural or physiological disturbances may be relevant to only subgroups of individuals within diagnostic categories. Using physiological or blood-based biomarkers to determine the functional status of the orexin system at an individual level may be helpful in identifying those individuals most likely to benefit from orexin-based medications.

## 9 | CONCLUSIONS

**FIGURE 3:**
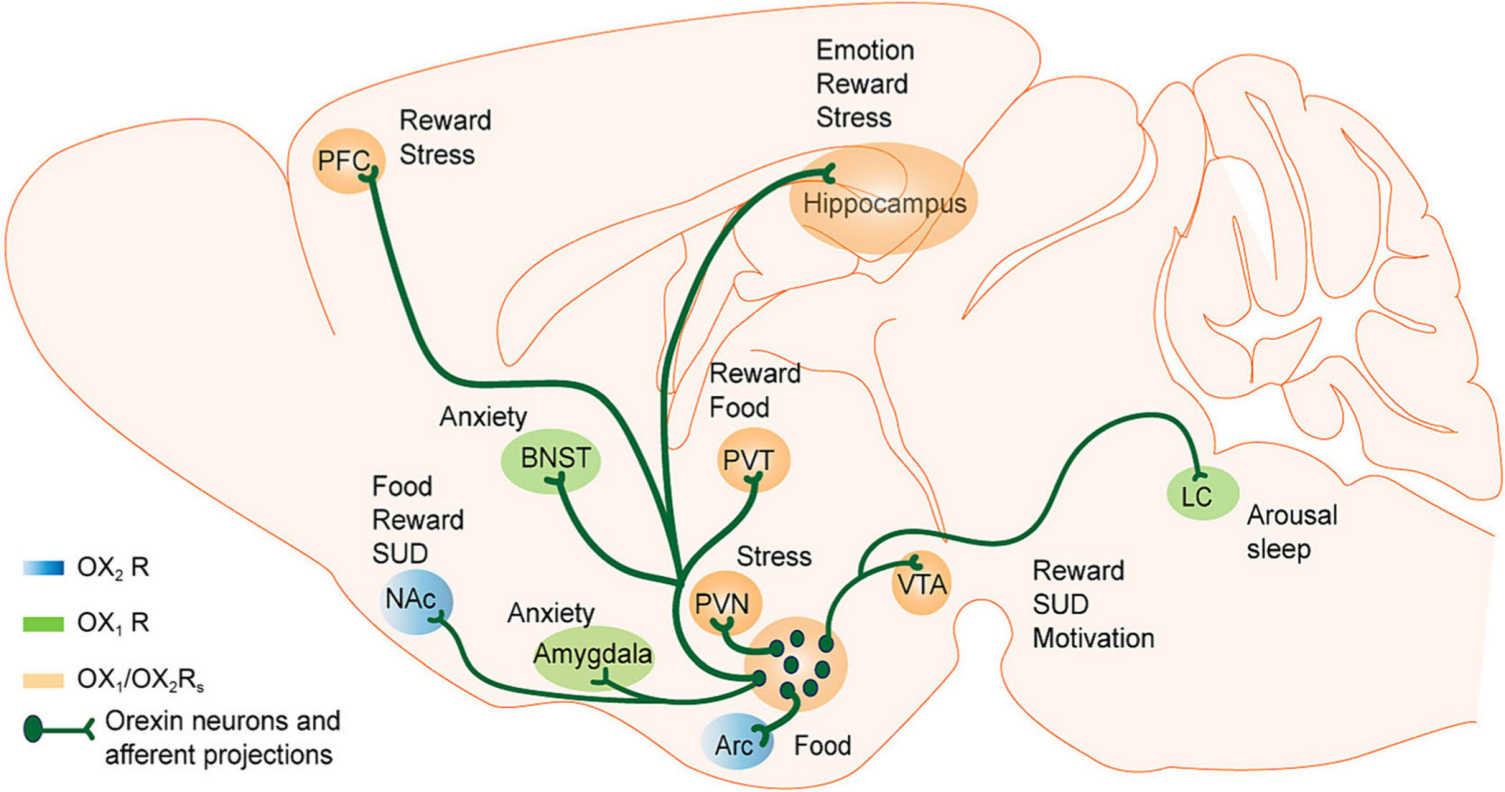
Schematic representation of the orexin neurocircuitry. Efferent projections of orexin/hypocretin neurons, the distribution of orexin receptors (OX_1_/OX_2_ receptors), and the associated behavioural functions in postsynaptic target regions. Orexin neurons project widely throughout the brain onto target regions including amygdala, arcuate nucleus (Arc), bed nucleus of the stria terminalis (BNST), hippocampus, nucleus accumbens (NAc), prefrontal cortex (PFC), paraventricular hypothalamic nucleus (PVN), paraventricular thalamic nucleus (PVT), locus coeruleus (LC) and ventral tegmental area (VTA). Areas with OX_1_ receptor distribution are shown in green, areas with OX_2_ receptor distribution are shown in blue, and areas with distribution of both OX_1_ and OX_2_ receptors are shown in orange. Abbreviation: R, receptor.

Over the past 25 years, significant progress has been made to understand the contributions of the orexin system to the certain disease pathophysiologies. It is now clear that the orexin system plays a central role in a variety of physiological processes such as arousal, feeding, sleeping, energy expenditure and reward seeking. Moreover, the behavioural output of orexin signalling is mediated by parallel signalling through interaction of multiple brain regions and signalling through orexin receptors (Figure 3). Behavioural effects are primarily mediated by OX_1_ receptors, including motivation for food and drugs, however more work is required to understand the potential contributions of OX_2_ receptors. Upregulation or downregulation of orexin system signalling can lead to changes in behaviour that mimic distinct neuropsychiatric conditions including anxiety-like, depression-like and addiction-like behaviours. The orexin system is anatomically positioned to regulate complex behaviours through its connections with neighbouring hypothalamic structures such as the stress-sensitive PVN the reward-related VTA, NAc and the prefrontal cortex. Through interactions with molecules and hormones such as glucose, ghrelin and leptin, orexin can modulate behaviours associated with feeding, including binge-eating behaviour. Accumulating evidence indicates that the widespread orexin innervation and multiple functional roles imply that this system plays a broad role in the central nervous system functions that integrate arousal with motivated behaviour. Mahler et al. (2014) proposed that the general role of the orexin system is the ability to activate and coordinate adaptive motivational responses to environmental as well as interoceptive signals, a role they termed motivational activation. In this view, orexin signalling transformed motivationally relevant states into adaptive behaviour directed towards exploiting an opportunity or managing a threat.

Despite substantial progress in understanding orexin neurons since their discovery, the rapidly evolving field of neuroscience (Duffet, Kosar, et al., 2022; Duffet, Tatarskiy, et al., 2022) holds promise for addressing remaining questions about this multitasking neuropeptide. From our view, the major priority for the field going forward is to explore the contribution of orexin inputs to brain regions implicated in motivated behaviour using novel technologies to selectively activate or inactivate these inputs in a temporally controlled manner. This ongoing research will undoubtedly deepen our comprehension of the orexin system role in regulating motivated behaviours and its consequential implications for various pathologies.

### 9.1 | Nomenclature of targets and ligands

Key protein targets and ligands in this article are hyperlinked to corresponding entries in the IUPHAR/BPS Guide to PHARMACOLOGY http://www.guidetopharmacology.org and are permanently archived in the Concise Guide to PHARMACOLOGY 2023/24 (Alexander, Christopoulos, et al., 2023; Alexander, Fabbro, et al., 2023; Alexander, Mathie, et al., 2023).

## Data Availability

N/A-Review.

## Abbreviations:

HPA axis: hypothalamus-pituitary-adrenal axis
LH: lateral hypothalamus
NAc: nucleus accumbens
NAcSH: nucleus accumbens shell
PVN: paraventricular nucleus
PVT: paraventricular thalamus
VTA: ventral tegmental area
